# Exosomes from senescent epithelial cells activate pulmonary fibroblasts via the miR-217-5p/Sirt1 axis in paraquat-induced pulmonary fibrosis

**DOI:** 10.1186/s12967-024-05094-x

**Published:** 2024-03-26

**Authors:** Min Zhang, Xiang Xue, Zhenshuai Lou, Yanhong Lin, Qian Li, Changbao Huang

**Affiliations:** Department of Emergency Medicine, The First Affiliated Hospital of Wannan Medical College (Yijishan Hospital of Wannan Medical College), Wuhu, 241001 Anhui People’s Republic of China

**Keywords:** Paraquat, Exosome, SIRT1, miR-217-5p, Pulmonary fibrosis

## Abstract

**Background:**

Paraquat (PQ) is a widely used and highly toxic herbicide that poses a significant risk to human health. The main consequence of PQ poisoning is pulmonary fibrosis, which can result in respiratory failure and potentially death. Our research aims to uncover a crucial mechanism in which PQ poisoning induces senescence in epithelial cells, ultimately regulating the activation of pulmonary fibroblasts through the exosomal pathway.

**Methods:**

Cellular senescence was determined by immunohistochemistry and SA-β-Gal staining. The expression of miRNAs was measured by qPCR. Pulmonary fibroblasts treated with specific siRNA of SIRT1 or LV-SIRT1 were used to analysis senescent exosomes-mediated fibroblasts activation. Luciferase reporter assay and western blot were performed to elucidated the underlying molecular mechanisms. The effects of miR-217-5p antagomir on pulmonary fibrosis were assessed in PQ-poisoned mice models.

**Results:**

Impairing the secretion of exosomes effectively mitigates the harmful effects of senescent epithelial cells on pulmonary fibroblasts, offering protection against PQ-induced pulmonary fibrosis in mice. Additionally, we have identified a remarkable elevation of miR-217-5p expression in the exosomes of PQ-treated epithelial cells, which specifically contributes to fibroblasts activation via targeted inhibition of SIRT1, a protein involved in cellular stress response. Remarkably, suppression of miR-217-5p effectively impaired senescent epithelial cells-induced fibroblasts activation. Further investigation has revealed that miR-217-5p attenuated SIRT1 expression and subsequently resulted in enhanced acetylation of β-catenin and Wnt signaling activation.

**Conclusion:**

These findings highlight a potential strategy for the treatment of pulmonary fibrosis induced by PQ poisoning. Disrupting the communication between senescent epithelial cells and pulmonary fibroblasts, particularly by targeting the miR-217-5p/SIRT1/β-catenin axis, may be able to alleviate the effects of PQ poisoning on the lungs.

**Supplementary Information:**

The online version contains supplementary material available at 10.1186/s12967-024-05094-x.

## Background

Paraquat (PQ) is widely used in field weed control due to its fast-acting and non-selective properties [1]. Despite the limitation imposed by numerous nations, the incidence of PQ self-poisoning continues to be widespread. In the case of France, the implementation of the ban in 2007 did not result in a decrease in the number of suicide attempts [2]. Similarly, in Korea, approximately 20% of farmers still persist in using PQ [3]. Despite the Chinese government's prohibition on the sale and utilization of PQ in 2016, isolated incidents of poisoning continue to arise [4]. To date, there is a dearth of efficacious treatments for PQ poisoning, leading to a significant mortality rate ranging from 60 to 80% [5]. PQ accumulates in the lungs and results in respiratory failure, which is the primary factor contributing to mortality caused by PQ exposure [1]. This accumulation causes acute lung injury which subsequently advances to pulmonary fibrosis, resulting in the irreversible loss of lung tissue [6]. Nevertheless, the precise mechanism by which PQ induces pulmonary fibrosis remains incompletely understood.

Excessive proliferation of fibroblasts surrounding the alveoli in the lungs is a prevalent characteristic of pulmonary fibrosis [7]. The aberrantly activated pulmonary fibroblasts are considered as an essential event driving pulmonary fibrogenesis [8]. Fibroblasts play a pivotal role in the synthesis and secretion of proteins, which are essential for the formation of the extracellular matrix (ECM). The ECM provides the normal scaffold for lung epithelium and endothelium, which are essential for efficient gas exchange and the repair of damaged tissue [9]. Many pro-fibrotic factors can stimulate fibroblast activation, resulting in an increased synthesis of ECM [10]. Previously, our study provided evidence that exposure to PQ can induce cellular senescence in epithelial cells, thereby facilitating the activation of pulmonary fibroblasts [11]. In fibroblastic foci, lung epithelial cells located in close proximity to myofibroblasts play a pivotal role in the initiation and progression of fibrotic processes [12]. These cells exhibit cellular senescence, along with atypical morphology and gene-expression profiles [13]. Senescent epithelial cells have been observed to generate a diverse array of growth factors, cytokines, and proteinases, which in turn promote the migration, proliferation, and activation of fibroblasts [14]. This evidence indicates that senescent epithelial cells have a substantial impact on the pathogenesis of pulmonary fibrosis.

Extracellular vesicles (EVs), specifically exosomes and microvesicles, have emerged as a novel paracrine mediator facilitating the transfer of various biological components. Notably, microRNAs (miRNAs) are among the cargo transported by EVs [15]. These miRNAs play a crucial role in gene regulation at both the transcriptional and posttranscriptional levels by binding to the 3'-untranslated regions (UTRs) of target mRNAs [16]. Exosomes are increasingly recognized as novel mediators of cell-to-cell communication and potential biomarkers for diverse lung diseases [17]. Exosomes, which are present in various biological fluids such as sputum, mucus, blood, urine, and bronchoalveolar lavage fluid (BALF), serve as valuable resources for studying the underlying mechanisms of lung diseases. They have been shown to play a crucial role in the development of pulmonary fibrosis. [18].

In this study, we have provided evidence to support the notion that the exosomes derived from senescent epithelium in lung tissues of PQ-poisoned mice exhibited pro-fibrotic effects via triggering pulmonary fibroblasts activation. We identified miR-217-5p was significantly elevated in the exosomes of PQ-treated epithelial cells, which promoted fibroblasts activation via targeted inhibition of the expression of SIRT1. Notably, suppression of miR-217-5p effectively impaired senescent epithelial cells-induced fibroblasts activation. Further mechanism studies revealed that miR-217-5p attenuated SIRT1 expression and subsequently resulted in enhanced acetylation of β-catenin and Wnt signaling activation. Our studies collectively propose a potential therapeutic approach for the management of pulmonary fibrosis induced by PQ by disrupting the communication between senescent epithelial cells and pulmonary fibroblasts.

## Materials and methods

### Ethical approval

This study was conducted in strict adherence to the guidelines outlined in the Guide for the Care and Use of Laboratory Animals provided by the National Institutes of Health. The protocol received approval from the Experimentation Ethics Review Committee of Wannan Medical College, with the ethics approval number IACUC-2017089. All surgical procedures were conducted using sodium pentobarbital anesthesia, with utmost care taken to minimize any potential discomfort or distress experienced by the subjects.

### Animal models and treatment

C57BL/6 mice, weighing between 18 and 22 g, were obtained from Hangzhou Ziyuan Animal Center (Hangzhou, China). The mice were housed in a controlled environment with regulated temperature and humidity, and subjected to a 12 h dark and 12 h light cycle. For the induction of pulmonary fibrosis [19], mice were administered intraperitoneal injections of either 20 mg/kg PQ in 100 μl saline (n = 6) or an equivalent volume of saline as a vehicle control (n = 6). For the purpose of treating PQ-induced pulmonary fibrosis, mice were administered GW4869 (2.5 mg/kg) via intraperitoneal injection twice a week [20], or received intratracheal injection of miR-217-5p antagomir on day 3 following PQ treatment. The lung tissues were collected on day 21 after PQ treatment for subsequent investigation. To examine the impact of exosomes obtained from senescent epithelial cells on pulmonary fibrosis, a group of mice was subjected to tracheal instillation with a dosage of 25 μg of exosomes in 50 μl of saline solution. At the 21st day, the lung tissues were collected for further investigation.

### Fibroblast isolation and culture

The pulmonary fibroblasts were obtained from the lung tissues of C57BL/6 mice, following the methodology described in a previous study [21]. Briefly, the lung tissues were dissected into small fragments measuring 1 mm^3^ and subsequently placed in DMEM medium supplemented with 0.2% collagenase I (Sigma-Aldrich). The fragments were then incubated at a temperature of 37 ℃ for a duration of 40 min. Next, the digested sample was filtered through a 40 μm-filter, and subsequently, the solution was rinsed three times with PBS. Subsequently, the cells were suspended in 10 ml of Dulbecco’s Modified Eagle Medium (DMEM) supplemented with 10% fetal bovine serum (FBS), and serially passaged until they achieved a confluence of 90%.

### Co-culture experiments

To examine the intercommunication between senescent epithelial cells and fibroblasts, a 6-well trans-well apparatus with a pore size of 0.4 μm (Costar, Cambridge, MA) was employed to establish an indirect co-culture system. Fibroblasts were introduced into the lower chamber, while PQ-pretreated MLE-12 cells or MLE-12 cells transfected with CD63-GFP were introduced into the upper chamber. The fibroblasts underwent additional analysis 48 h after co-culturing.

### Exosome isolation

The exosomes were obtained through the process of differential centrifugation [22]. Mouse bronchoalveolar lavage fluid (BALF) and serum-free cell culture media were collected and subjected to centrifugation at 300×g for 5 min to eliminate dead cells, followed by centrifugation at 3000×g for 10 min to remove cellular debris. The supernatant was subjected to centrifugation at 12000×g for 30 min, followed by ultracentrifugation at 100000×g for 70 min, in order to isolate the exosomes. The sizes and concentrations of exosomes were assessed using a NanoSight NS300 instrument manufactured by Malvern Instruments. The morphology of the sample was assessed using transmission electron microscopy (TEM). Furthermore, the quantification of exosomes was performed using the BCA assay provided by Thermo Fisher.

### siRNA, miRNA and lentivirus transfection

Fibroblasts were transfected with SIRT1-siRNA, miR-217-5p mimic (50 nM), miR-217-5p inhibitor (100 nM), or the corresponding negative control according to the manufacturer’s instruction (RIBOBIO, Guangzhou, China). The determination of transfection efficiency was conducted using quantitative polymerase chain reaction (qPCR). In order to enhance the expression of SIRT1, fibroblasts were transfected with a lentiviral vector containing a gene sequence encoding for SIRT1 at a concentration of 1 × 10^7^ TU/mL.

### qPCR

Total RNA was extracted from cells or lung tissues using TRIzol (Invitrogen). The concentration of the purified RNA was determined using a NanoDrop Lite spectrophotometer (Thermo Scientific). Complementary DNAs (cDNAs) were synthesized using the HiScript 1st Strand cDNA Synthesis Kit (Vazyme, Nanjing, China). Quantitative polymerase chain reaction (qPCR) with SYBR Green was conducted to assess the expression of specific messenger RNAs (mRNAs), which were normalized to the expression of GAPDH. For the detection of microRNAs (miRNAs) in exosomes, the miRNAs were reverse transcribed using specific neck loop primers and quantified using quantitative polymerase chain reaction (qPCR). The quantification of miRNA copy numbers in exosomes was performed using the absolute quantification method [23], and the obtained results were subsequently normalized to the number of vesicles determined using a reference control. All primer sequences are provided in Additional file 1: Table 1.

### Histological examination

Mouse lung tissues were subjected to fixation using 10% neutral formalin prior to being embedded in paraffin wax through standard procedures. The lung tissues were sectioned into 5 μm thick slices in order to perform hematoxylin–eosin (H&E), Masson trichrome, and immunohistochemical staining. For the immunohistochemical (IHC) analysis, the lung sections were immersed in a solution of 0.3% hydrogen peroxide in methanol for a duration of 20 min in order to eliminate any endogenous peroxidase activity. Subsequently, the sections were treated with goat serum for a period of 1 h at room temperature to inhibit any nonspecific binding of immunoglobulin. Tissue sections were incubated overnight with primary antibodies. Finally, the lung sections underwent a PBST wash and were subsequently incubated with the appropriate secondary antibody for a duration of 1 h. The visualization of antigen-positive cells was achieved using the DAB Substrate kit (ZSGB-BIO, Beijing, China).

### Immunofluorescence staining

Fibroblasts were rinsed with PBS prior to fixation using a 4% paraformaldehyde solution. For the detection of lung tissues, the sections underwent deparaffinization in xylene, followed by hydration with decreasing concentrations of ethanol. Following that, the cells or lung sections were rinsed with PBST and subsequently treated with 0.3% Trion X-100 to enhance permeability. Samples were subsequently blocked with goat serum for a duration of 1 h at room temperature, followed by incubation with primary antibodies overnight at a temperature of 4 °C. Subsequently, the samples were incubated in darkness with fluorescent-labeled secondary antibodies for a duration of 1 h. Finally, the nuclei were stained with DAPI (Santa Cruz). Images were acquired using an Olympus confocal laser microscope.

### Western blot

Cells or lung tissues were homogenized using RIPA lysis buffer (KeyGEN BioTECH, Nanjing, China). Subsequently, the protein extracts were separated using SDS-polyacrylamide gel electrophoresis and subsequently transferred onto PVDF membranes. The membranes were incubated with the specified primary antibodies overnight following the blocking step with 5% BSA. Blots were developed using HRP-conjugated secondary antibodies provided by Abclonal. Protein bands were visualized using an enhanced chemiluminescence (ECL) solution (Thermo) and detected using an automated chemiluminescence imaging analysis system (Tanon, Shanghai, China).

### 3′UTR-luciferase reporter constructs

The wild-type or mutant 3' untranslated regions (UTRs) of SIRT1, which included the anticipated binding site for miR-217-5p, were synthesized and inserted into the pGL3.0-control vectors following the guidelines provided by the manufacturer (OBIO, Shanghai, China). Fibroblasts were seeded in 96-well plates at a density of 1 × 10^5^ cells per well. Cells were transfected with 10 pmol of miR-217-5p mimics or scramble controls (RIBOBIO, Guangzhou, China) and co-transfected with 0.2 μg per well of wild-type SIRT1 3′UTR-luc or mutant SIRT1 3′UTR-luc. Following a 48 h transfection period, the cells were lysed, and the luciferase activities were quantified using a dual-luciferase assay kit provided by Promega.

### Hydroxyproline content of whole lung

To quantify collagen levels in lung tissues, the Hydroxyproline Colorimetric Assay kit (BioVision, Milpitas, CA) was employed for the hydroxyproline assay. Briefly, mouse lung tissues weighing 10 mg were homogenized in 100 μl of hydrochloric acid (HCl, 12 N) and subjected to hydrolysis at a temperature of 120 °C for a duration of 3 h. Subsequently, 10 μl of each individual sample were utilized to measure the absorbance at a wavelength of 560 nm. The concentration of hydroxyproline in each sample was determined by comparing it to the quantity of lung tissue utilized.

### Statistics and analysis

All statistical analyses were conducted utilizing GraphPad Prism (San Diego, CA) or SPSS 25.0 (IBM, Armonk, NY). Data analysis was conducted using analysis of variance (ANOVA) followed by the Tukey post hoc test for parametric data, or the Kruskal–Wallis test followed by Dunn’s test for nonparametric data. The data are presented in the form of the mean ± standard error of the mean (SEM). In all instances, a significance level of p < 0.05 was employed to determine statistical significance.

## Results

### PQ promotes epithelial cell senescence and exosome secretion in vivo and in vitro

**Fig. 1:**
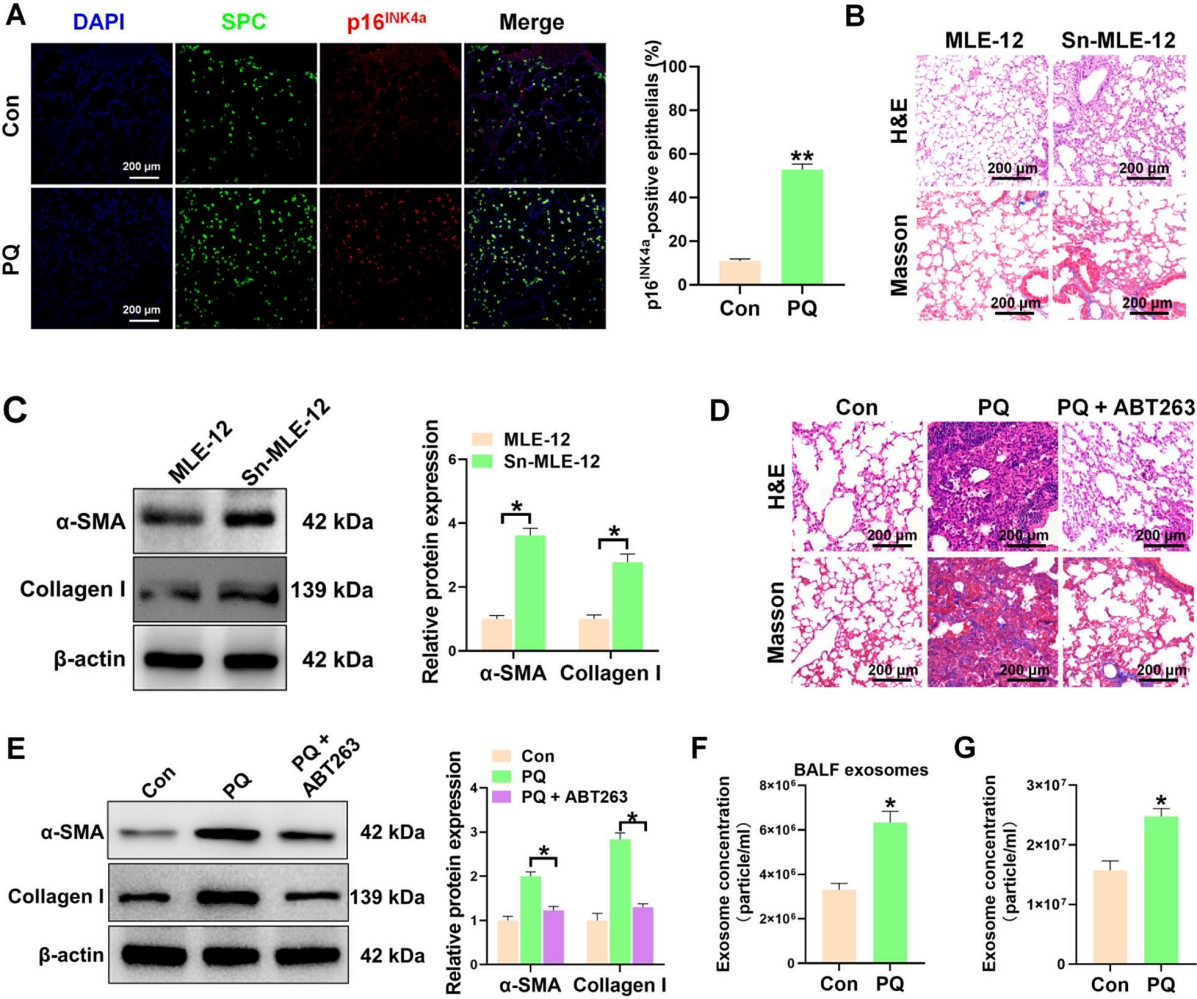
PQ promotes epithelial cell senescence and exosome secretion in vivo and in vitro. **A** Representative images and quantification of coimmunostaining of SPC (Green) and p16^INK4a^ (Red) in the lung tissues from the mice treated with or without PQ. n = 5 per group. **B** The representative H&E staining (up panel) and Mssson’s trichrome staining (down panel) images of the lung tissues from mice intratracheally injected with MLE-12 cells or senescent MLE-12 cells (Sn-MLE-12). n = 5 per group. **C** Western blot analysis of the expression of α-SMA and Collagen I in the lung tissues from mice intratracheally injected with MLE-12 cells or Sn-MLE-12 cells. **D** The representative H&E staining (up panel) and Masson’s trichrome staining (down panel) images of the lung tissues from PQ-poisoned mice treated with or without ABT263. n = 5 per group. **E** Western blot analysis of the expression of α-SMA and Collagen I in the lung tissues from PQ-poisoned mice treated with or without ABT263. **F** Concentration of exosomes from BALF of mice treated with or without PQ. n = 5 per group. **G** The concentration of exosomes in the supernatant of MLE-12 cells treated with or without PQ. n = 3 per group. All data were presented as the means ± SEM. *P < 0.05

Recently, growing evidence demonstrated that cellular senescence played an essential role in the progression of pulmonary fibrosis [24]. To investigate the involvement of cellular senescence in PQ-induced pulmonary fibrosis, the expression of p16^INK4a^ a typical mark of cellular senescence was measured in the lung tissues of mice exposed to PQ poisoning. Our findings revealed a significant elevation in the presence of senescent alveolar epithelial cells within the lung tissues of mice treated with PQ (Fig. 1A). In addition, a significant number of SA-β-Gal positive cells were observed in vitro in PQ-treated MLE-12 cells (Additional file 1: figure S1A). In vivo, intratracheal injection of PQ-induced senescent MLE-12 cells dramatically increased the extent of lung damage and collagen deposition (Fig. 1B), accompany with enhanced levels of fibrotic markers (Fig. 1C). To further confirm the role of senescent epithelial cells in the progression of PQ-induced pulmonary fibrosis, mice poisoned with PQ were treated with ABT263, a potent senolytic drug that selectively and rapidly induces the apoptosis of senescent cells. Our results showed that clearance of senescent cells by ABT263 could dramatically impair PQ-induced pulmonary fibrosis (Fig. 1D, E). The findings of this study suggest that the process of epithelial cell senescence plays a significant role in the development of pulmonary fibrosis induced by PQ.

In our previous study, we demonstrated that the secretome of PQ-treated epithelial cells exhibited fibrotic properties, which could potentially stimulate the activation of pulmonary fibroblasts in vitro. Exosomes as secreted nanoparticles by all cell types are important components in intercellular communication, which could carry biologic factors from one cell type or tissue to another [15]. Interestingly, we found that the expression of CD63 was dramatically elevated in the lung tissues of PQ-poisoned mice (Additional file 1: figure S2). We observed elevated numbers of exosomes in the BALF obtained from PQ-treated mice (Fig. 1F). In vitro, PQ profoundly increased CD63 levels in MLE-12 cells (Additional file 1: figure S3). In addition, we isolated and purified exosomes from the conditioned medium of MLE-12 cells treated with or without PQ. The number and size of isolated exosome were determined by nanosight particle tracking analysis (Fig. 1G, Additional file 1: figure S4), which showed that the secretion of exosomes was also enhanced in PQ-treated MLE-12 cells (Fig. 1G). These results indicated that aberrant exosomes secretion of senescent epithelial cells may play an essential role in epithelial senescence-mediated fibrogenesis.

### Senescent epithelial cells induce fibroblast activation through exosome secretion

**Fig. 2:**
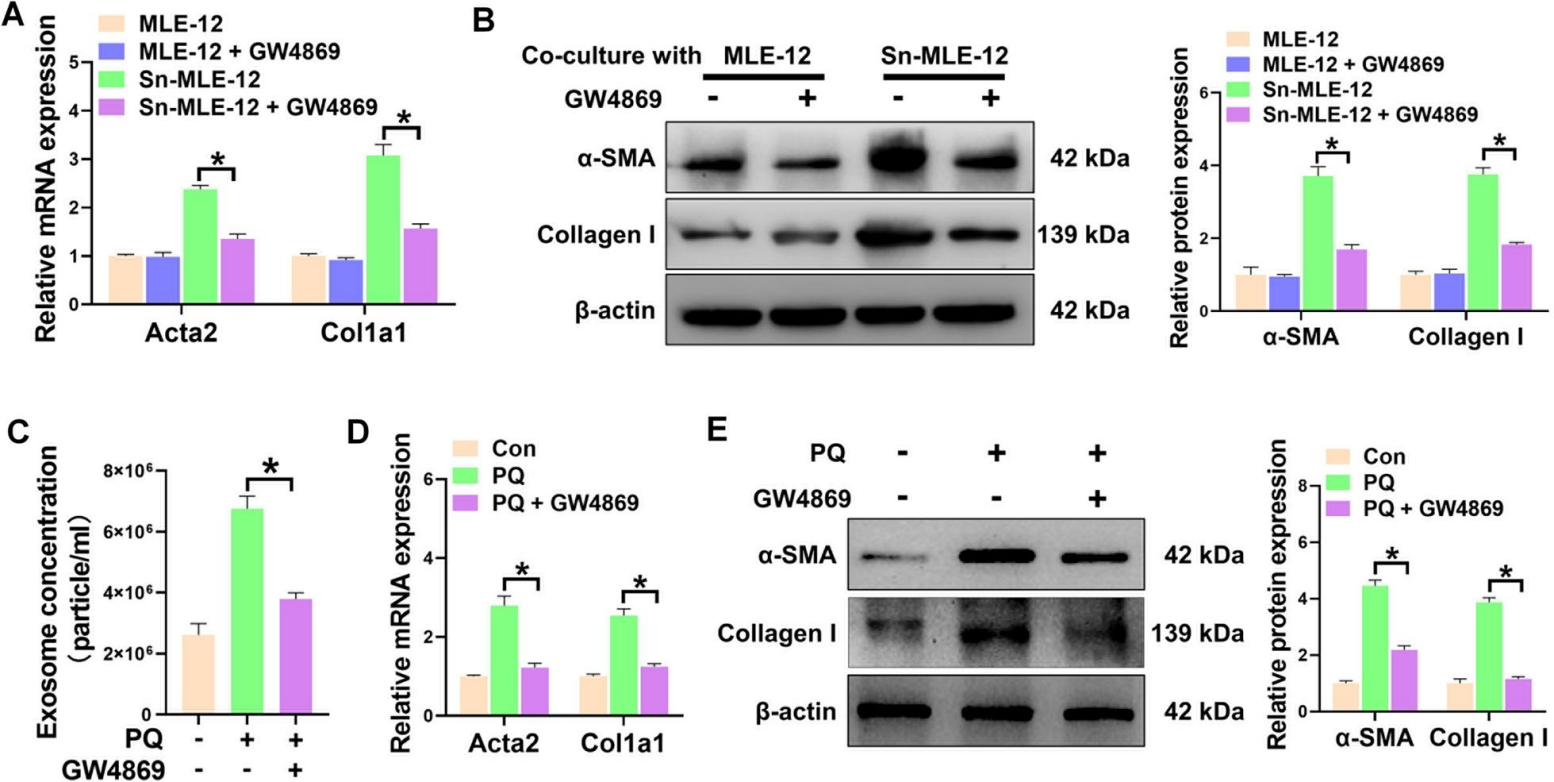
Senescent epithelial cells induce fibroblast activation through exosome secretion. **A** qPCR analysis of the mRNA levels of Acta2 and Col1a1 in pulmonary fibroblasts co-cultured with GW4869-pretreated MLE-12 cells or senescent MLE-12 cells (Sn-MLE-12). **B** Western blot analysis of the expression of α-SMA and Collagen I in pulmonary fibroblasts co-cultured with GW4869-pretreated MLE-12 cells or Sn-MLE-12 cells. **C** Concentration of exosomes from BALF of PQ-poisoned mice injected with or without GW4869. n = 5 per group. **D**, **E** qPCR and Western blot analysis of the mRNA (**D**) and protein (**E**) levels of α-SMA and Collagen I in the lung tissues from PQ-poisoned mice injected with or without GW4869. All data were presented as the means ± SEM. *P < 0.05

To verify whether senescent epithelial cells-induced fibroblast activation was mediated through exosome secretion, senescent MLE-12 cells were pretreated with GW4869, a noncompetitive inhibitor of N-SMase, to block exosome secretion. Then, senescent MLE-12 cells were cocultured with pulmonary fibroblasts. The levels of ∝-SMA and Collagen I expression were found to be significantly reduced in pulmonary fibroblasts that were co-cultured with GW4869-pretreated senescent MLE-12 cells, in comparison to cells that were co-cultured with senescent MLE-12 cells. (Fig. 2A, B), suggesting that the profibrotic effects of senescent epithelial cells were dramatically abolished after GW4869 treatment. Next, we investigated whether blocking exosome secretion could suppress the progression of PQ-induced pulmonary fibrosis. PQ-poisoned mice were treated with either saline or GW4869. We observed a significant reduction in the number of exosomes in the BALF obtained from PQ-poisoned mice treated with GW4869, compared to PQ-poisoned mice (Fig. 2C). Furthermore, the H&E and Masson’s trichrome staining demonstrated that blocking exosome secretion effectively mitigated the progression of pulmonary fibrosis following PQ poisoning (Additional file 1: figure S5). The protein and mRNA expression levels of fibrotic gene were also significantly lower in PQ-poisoned mice treated with GW4869 than treated with saline (Fig. 2D, E). Collectively, our data demonstrated that inhibiting the release of exosomes following PQ poisoning can provide significant protection against PQ-induced pulmonary fibrosis in mice.

### Senescent epithelial cell-derived Exosomes promote the activation of fibroblast

**Fig. 3:**
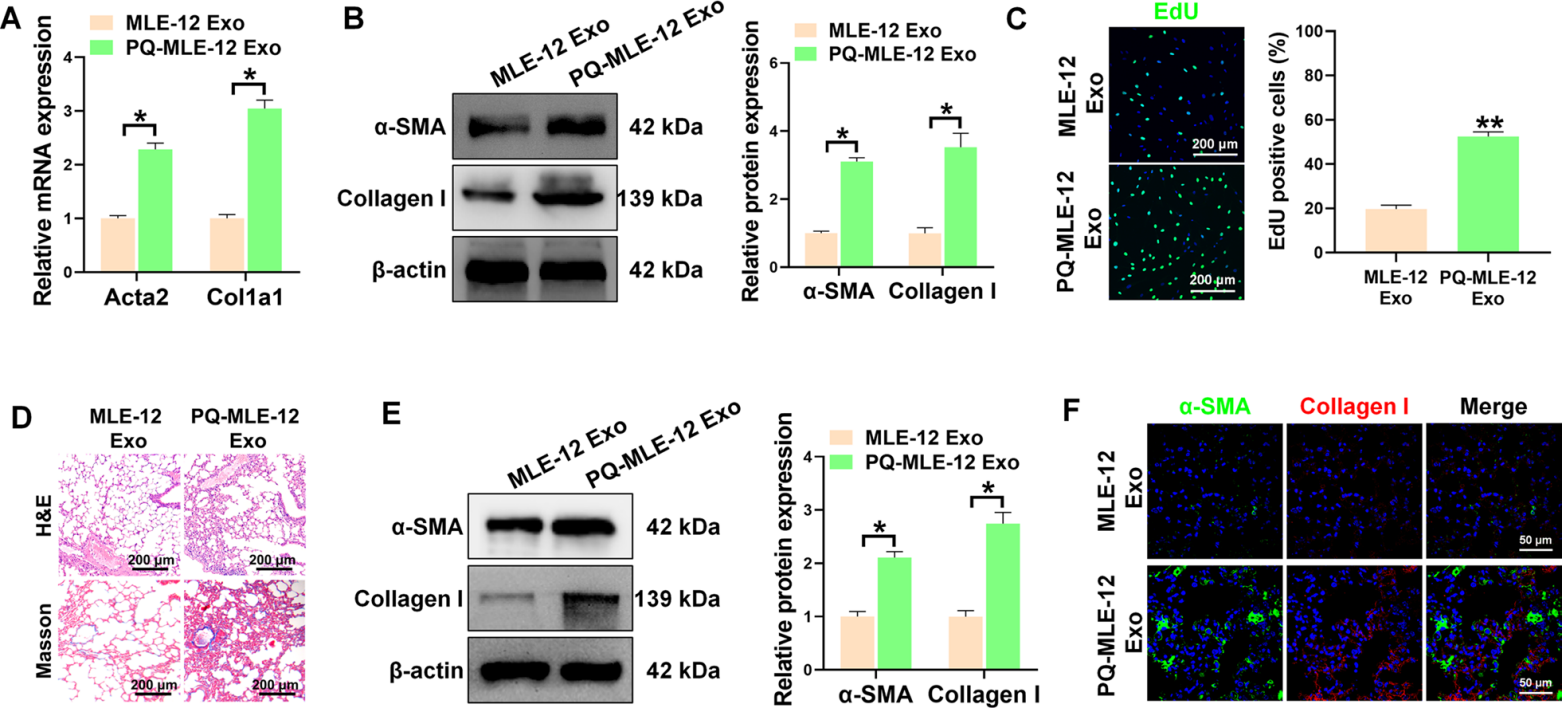
Senescent epithelial cell-derived Exosomes promote the activation of fibroblast. **A**, **B** The mRNA and protein levels of α-SMA and Collagen I in pulmonary fibroblasts treated with the exosomes isolated from MLE-12 cells (MLE-12 Exo) or PQ-treated MLE-12 cells (PQ-MLE-12 Exo). **C** The EdU assay of pulmonary fibroblasts treated with MLE-12 Exo or PQ-MLE-12 Exo. **D** The representative H&E staining (up panel) and Masson’s trichrome staining (down panel) images of the lung tissues from mice intratracheally injected with MLE-12 Exo or PQ-MLE-12 Exo. n = 5 per group. **E**, **F** Western blot and immunofluorescent analysis of the expression of α-SMA (Green) and Collagen I (Red) in the lung tissues from mice intratracheally injected with MLE-12 Exo or PQ-MLE-12 Exo. All data were presented as the means ± SEM. *P < 0.05

To identify whether exosomes mediated the communication between senescent epithelial cell and fibroblasts, MLE-12 cells were transfected with GFP-labeled CD63 vectors before coculture with fibroblasts. Confocal imaging showed elevated GFP spots in recipient fibroblasts that co-cultured with PQ-treated MLE-12 cells (Additional file 1: figure S6). These results indicated that exosomes released by PQ-treated MLE-12 cells were delivered to fibroblasts. Next, we aimed to determine whether senescent epithelial cell-derived exosomes may contribute to the activated phenotype of fibroblasts. In vitro, fibroblasts were treated with isolated exosomes derived from MLE-12 cells treated with or without PQ. Our results showed that exosomes derived from PQ-treated MLE-12 cells promoted the differentiation of fibroblasts into myofibroblasts with elevated expression of fibrotic markers α-SMA and Collagen I (Fig. 3A, B). The Edu assay further confirmed that senescent epithelial cells-derived exosomes could remarkedly improve the proliferation ability of fibroblasts (Fig. 3C). In addition, intratracheal injection of exosomes obtained from PQ-treated epithelial cells markedly contributed to the deposition of collagen in pulmonary alveoli (Fig. 3D), accompany with enhanced myofibroblast differentiation in vivo (Fig. 3E, F). Altogether, these results indicated that exosomes derived from PQ-poisoned epithelial cells contributed to fibroblasts activation and promoted the pathogenesis of pulmonary fibrosis.

### MiR-217-5p in exosomes from senescent epithelial cells mediates fibroblasts activation

**Fig. 4:**
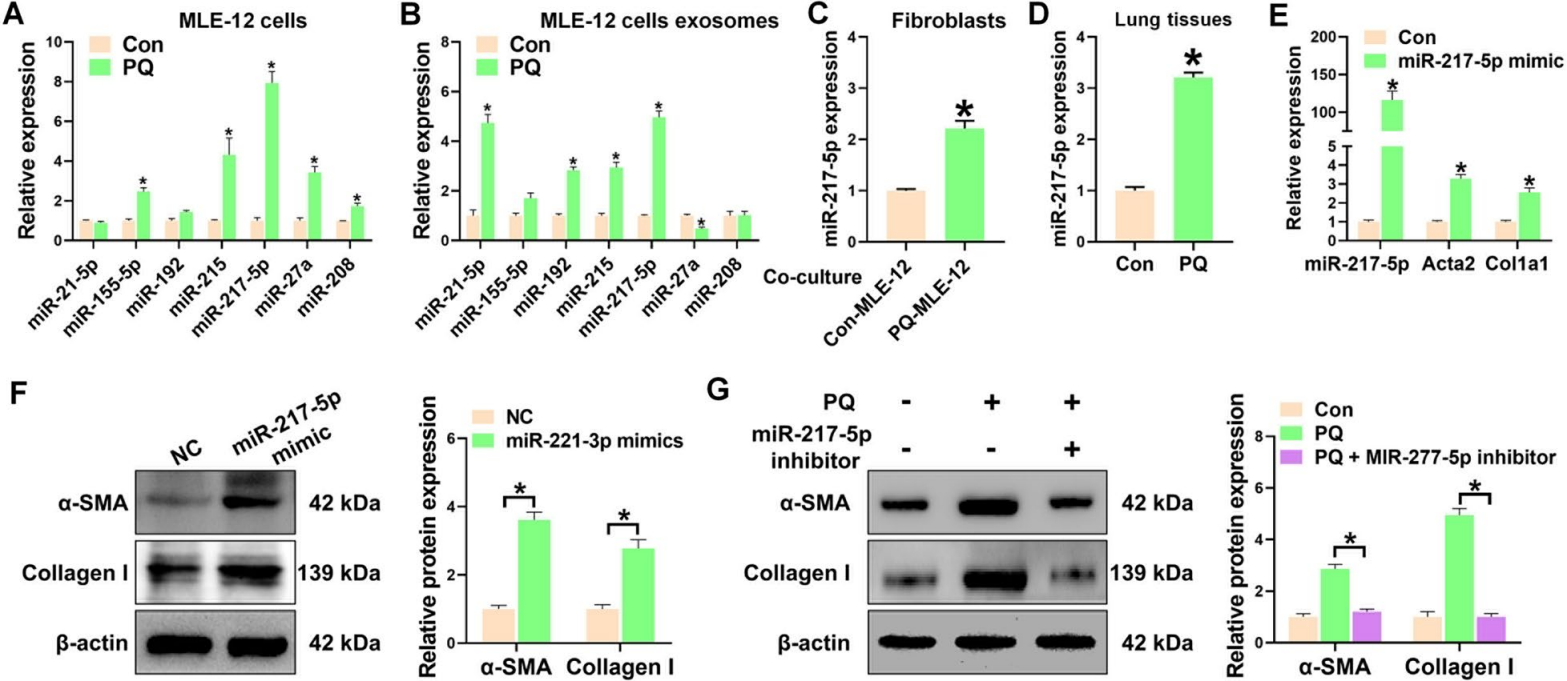
MiR-217-5p in exosomes from senescent epithelial cells mediates fibroblasts activation. **A** qPCR analysis of the miRNAs expression in MLE-12 cells treated with or without PQ. **B** qPCR analysis of the miRNAs expression in the exosomes isolated from MLE-12 cells treated with or without PQ. **C** qPCR analysis of miR-217-5p expression in pulmonary fibroblasts co-cultured with MLE-12 cells or PQ-treated MLE-12 cells. **D** qPCR analysis of miR-217-5p expression in the lung tissues from mice treated with or without PQ. **E** qPCR analysis of the expression of miR-217-5p, Acta2 and Col1a1 in pulmonary fibroblasts transfected with or without miR-217-5p mimic. **F** Western blot analysis of the expression of α-SMA and Collagen I in pulmonary fibroblasts transfected with or without miR-217-5p mimic. **G** Western blot analysis of the expression of α-SMA and Collagen I in pulmonary fibroblasts co-cultured with PQ-treated MLE-12 cells transfected with or without miR-217-5p inhibitor. n = 3 per group. All data were presented as the means ± SEM. *P < 0.05

We subsequently investigated the mechanism by which exosomes derived from senescent epithelial cells stimulate the activation of fibroblasts. The abundance and crucial role of miRNAs encapsulated in exosomes in facilitating communication between various cell types have been well-documented [25]. Therefore, we quantified the levels of pro-fibrotic miRNAs, such as miR-21-5p, miR-155-5p, miR-192, miR-215, miR-217-5p, miR-27a, and miR-208, in MLE-12 cells treated with PQ and in exosomes obtained from the culture medium of PQ-treated MLE-12 cells. Our findings demonstrated that both miR-217-5p and miR-215 were significantly increased in epithelial cells treated with PQ (Fig. 4A) and in exosomes derived from PQ-treated epithelial cells (Fig. 4B). In addition, miR-21-5p and miR-192 were also found to be elevated in exosomes derived from PQ-treated epithelial cells (Fig. 4B). To identify the miRNA that plays an essential role in fibroblast activation induced by the exosomes of PQ-treated epithelial cells, we further measured the expression of miR-217-5p and miR-215 in the fibroblasts that were co-cultured with PQ-treated MLE-12 cells. Our results showed that only miR-217-5p was significantly increased in fibroblasts that were co-cultured with PQ-treated MLE-12 cells (Fig. 4C, Additional file 1: figure S7). Moreover, the elevated expression of miR-217-5p was also observed in the lung tissue of mice exposed to PQ (Fig. 4E). In vitro, transfection of miR-217-5p mimic in fibroblasts remarkably elevated the expression of α-SMA and Collagen I (Fig. 4E, F), which indicated that activated fibroblasts may be a direct regulatory result of miR-217-5p. To provide additional evidence regarding the involvement of miR-217-5p in fibroblast activation mediated by senescent epithelial cells, MLE-12 cells treated with PQ were transfected with a miR-217-5p inhibitor. Interestingly, senescent epithelial cells-mediated upregulation of α-SMA and Collagen I in fibroblasts were dramatically attenuated when senescent epithelial cells transfected with miR-217-5p inhibitor (Fig. 4G). Collectively, these findings revealed that senescent epithelial cells-derived exosome miR-217-5p mediates the activation of fibroblasts.

### Senescent epithelial cells-derived exosome miR-217-5p directly targets SIRT1 in fibroblasts

**Fig. 5:**
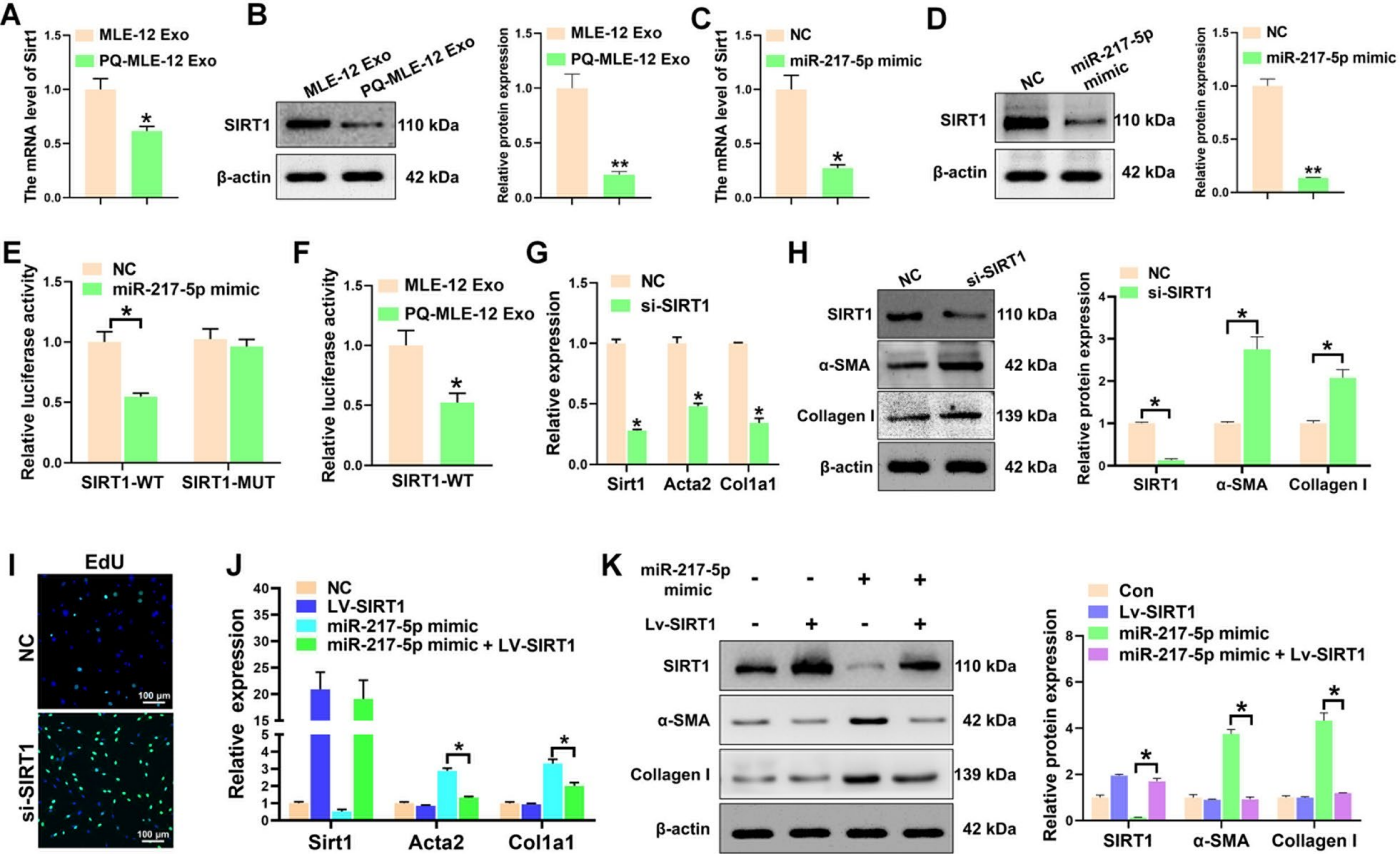
Senescent epithelial cells-derived exosome miR-217-5p directly targets SIRT1 in fibroblasts. **A** qPCR analysis of the mRNAs expression of Sirt1 in pulmonary fibroblasts treated with the exosomes isolated from MLE-12 cells (MLE-12 Exo) or PQ-treated MLE-12 cells (PQ-MLE-12 Exo). **B** Western blot analysis of SIRT1 expression in pulmonary fibroblasts treated with MLE-12 Exo or PQ-MLE-12 Exo. **C, D** qPCR (**C**) and Western blot (**D**) analysis of SIRT1 expression in pulmonary fibroblasts transfected with or without miR-217-5p mimic. **E** Relative luciferase activity analysis in pulmonary fibroblasts co-transfected with wide type (WT) or mutant type (MUT) SIRT1-3’UTR reporters together with miR-217-5p mimic or negative control (NC). **F** Relative luciferase activity analysis in SIRT1-WT-transfected pulmonary fibroblasts that were further treated with MLE-12 Exo or PQ-MLE-12. **G**, **H** qPCR (**G**) and Western blot (**H**) analysis of the expression of SIRT1, α-SMA and Collagen I in pulmonary fibroblasts transfected with or without SIRT1 siRNAs (si-SIRT1). **I** EdU assay for the proliferation of pulmonary fibroblasts transfected with or without si-SIRT1. **J**, **K** qPCR (**J**) and Western blot (**K**) analysis of the expression of SIRT1, α-SMA and Collagen I in pulmonary fibroblasts transfected with or without miR-217-5p mimic and co-transfected with or without LV-SIRT1. All data were presented as the means ± SEM. *P < 0.05, *P < 0.01

To identify the potential targets of miR-217-5p in fibroblasts, we performed target prediction using the databases TargetScan and miRDB, which showed that the seed region of miR-217-5p targets SIRT1 mRNA with complete complementarity (Additional file 1: figure S8). SIRT1, as a lysine deacetylase, participates in many physiological processes, which could attenuate fibroblasts activation and pulmonary fibrogenesis [26]. In vitro, the expression of SIRT1 was observed to be significantly reduced in fibroblasts that were treated with exosomes derived from PQ-poisoned MLE-12 cells, as evidenced by decreased levels of both mRNA and protein (Fig. 5A, B). Exogenous upregulation of miR-217-5p was found to have the ability to suppress the expression of SIRT1 (Fig. 5C, D), suggesting that SIRT1 could be a potential target of miR-217-5p. To provide additional evidence that SIRT1 is the specific target of miR-217-3p, we cloned both the wild-type and mutated versions of the miR-217-3p-binding site in the 3' UTR region of SIRT1 into the luciferase vector. Our findings indicate a significant decrease in luciferase activity in fibroblasts that were co-transfected with a wild-type binding site vector and a miR-217-5p mimic (Fig. 5E). However, fibroblasts containing the mutated binding site vector exhibited no significant repression of luciferase activity when transfected with miR-217-5p mimic (Fig. 5E). Similarly, the luciferase activity exhibited a significant reduction in fibroblasts that were transfected with the wild-type binding site vector of SIRT1, following the administration of exosomes derived from PQ-treated MLE-12 cells (Fig. 5F). The findings of this study suggest that miR-217-5p directly targets SIRT1 in fibroblasts.

To further determine the function of SIRT1 in miR-217-5p-mediated fibroblasts activation, fibroblasts were transfected with small interfering RNAs targeting SIRT1 (si-SIRT1) and the effect was verified by qPCR. As shown in Fig. 5G–H, downregulation of SIRT1 expression resulted in the profound elevation of fibrotic genes, accompany with enhanced proliferation ability of fibroblasts (Fig. 5I). In contrast, overexpression of SIRT1 could effectively neutralize the effect of miR-217-5p on fibroblasts activation (Fig. 5J, K). Collectively, these results suggested that miR-217-5p-mediated activation of fibroblasts is in a SIRT1 dependent manner.

### MiR-217-5p activates fibroblasts via SIRT1/β-catenin axis

**Fig. 6:**
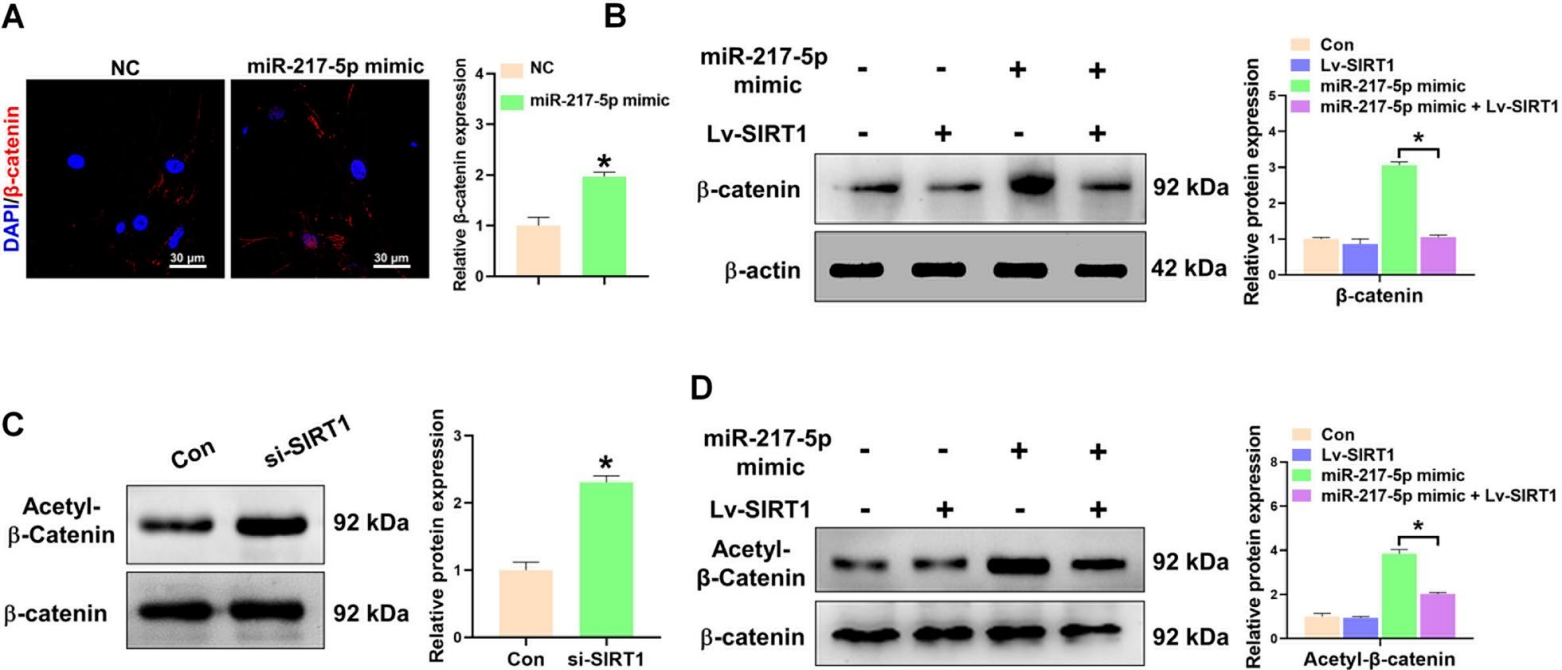
miR-217-5p activates fibroblasts via SIRT1/β-catenin axis. **A** Representative images and quantification of immunofluorescent detection for β-catenin (Red) in pulmonary fibroblasts transfected with or without miR-217-5p mimic. **B** Western blot analysis of the expression of β-catenin in pulmonary fibroblasts transfected with or without miR-217-5p mimic and co-transfected with or without LV-SIRT1. **C** Western blot analysis of the acetylation levels of β-catenin in pulmonary fibroblasts transfected with or without SIRT1 siRNA (si-SIRT1). **D** Western blot analysis of the acetylation levels of β-catenin in pulmonary fibroblasts transfected with or without miR-217-5p mimic and co-transfected with or without LV-SIRT1. All data were presented as the means ± SEM. *P < 0.05

It was documented that Wnt signaling is a highly conserved in fibrogenesis, the perturbation of Wnt signaling exhibited a direct correlation with the excessive activation of fibroblasts and the amplification of the fibrotic process [27]. Upregulation of miR-217-5p resulted in elevated expression and nuclear translocation of β-catenin (Fig. 6A), which suggested the activation of Wnt signaling. Therefore, to clarify the underlying regulation mechanism of SIRT1 and Wnt signaling in miR-217-5p-mediated fibroblasts activation, the expression of β-catenin was measured in fibroblasts co-transfected with miR-217-5p mimic and SIRT1. Our results showed that upregulation of SIRT1 could profoundly suppress miR-217-5p-mediated elevation of β-catenin in fibroblasts (Fig. 6B), which indicated that SIRT1 plays a crucial role in the activation of Wnt signaling. SIRT1, a histone deacetylase that depends on NAD + , is expressed throughout the body and has significant implications in the regulation of numerous diseases via modulation of the acetylation of various transcription factors and signaling proteins [28]. Inhibition of SIRT1 via si-SIRT1 could result in increased levels of acetylated β-catenin (Fig. 6C). In vitro, we demonstrated that the acetylation of β-catenin was significantly enhanced in fibroblasts treated with miR-217-5p mimic, which could be suppressed via upregulation of SIRT1 (Fig. 6D). These results suggested that SIRT1-modulated acetylation of β-catenin play an essential role in miR-217-5p-induced fibroblasts activation.

### Suppression of miR-217-5p ameliorates PQ-induced pulmonary fibrosis

**Fig. 7:**
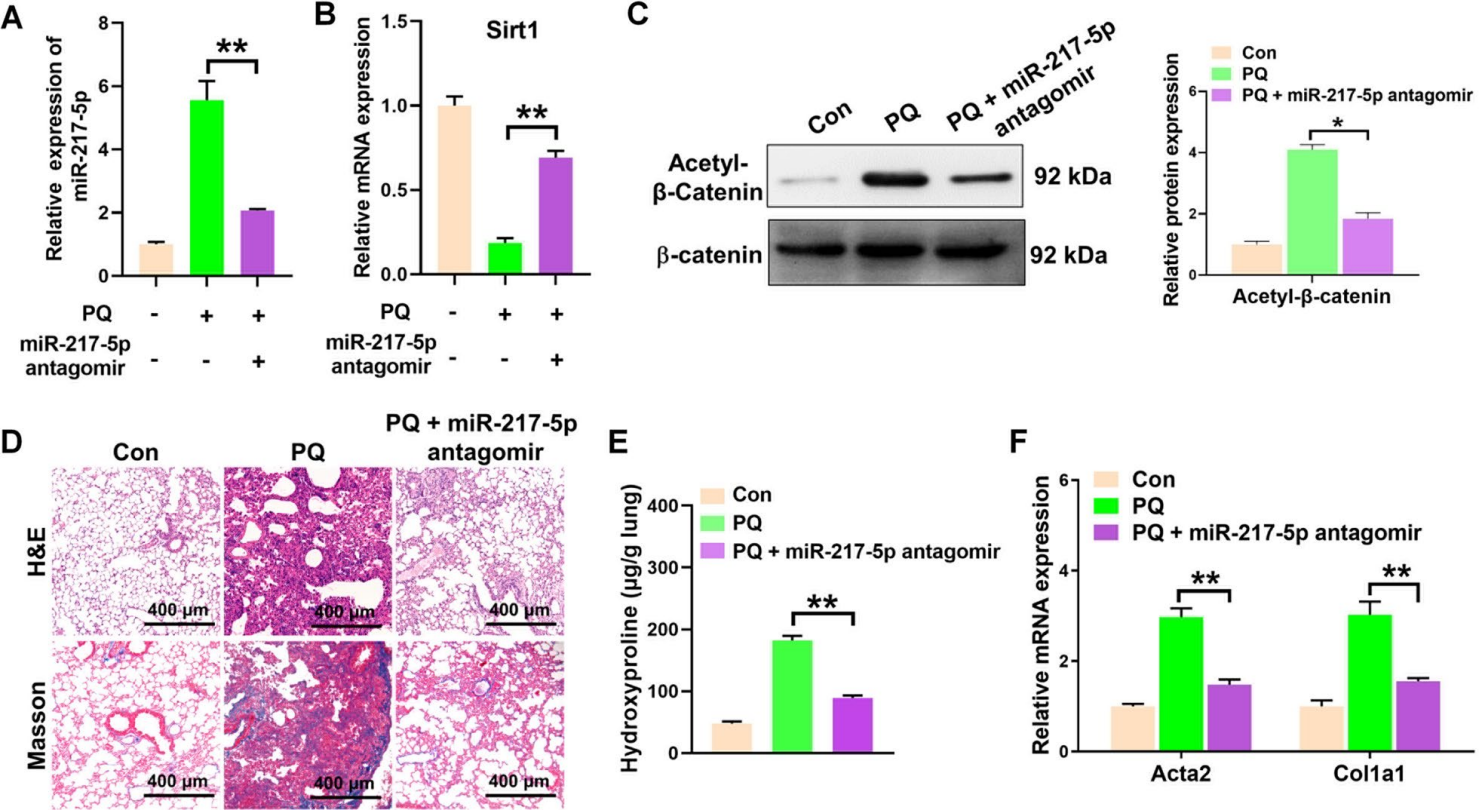
Suppression of miR-217-5p ameliorates PQ-induced pulmonary fibrosis. **A**, **B** qPCR analysis of the expression of miR-217-5p **A** and Sirt1 **B** in the lung tissues from PQ-poisoned mice with or without the treatment of miR-217-5p antagomir. n = 5 per group. **C** Western blot analysis of the acetylation levels of β-catenin in in the lung tissues from mice treated as in (**A**). **D** The representative H&E staining (up panel) and Masson’s trichrome staining (down panel) images of the lung tissues from mice treated as in (**A**). **E** The analysis of hydroxyproline in the lung tissues from mice treated as in (**A**). **F** qPCR analysis of the expression of Acta2 and Col1a1 in the lung tissues from mice treated as in (**A**). All data were presented as the means ± SEM. **P < 0.01

**Fig. 8:**
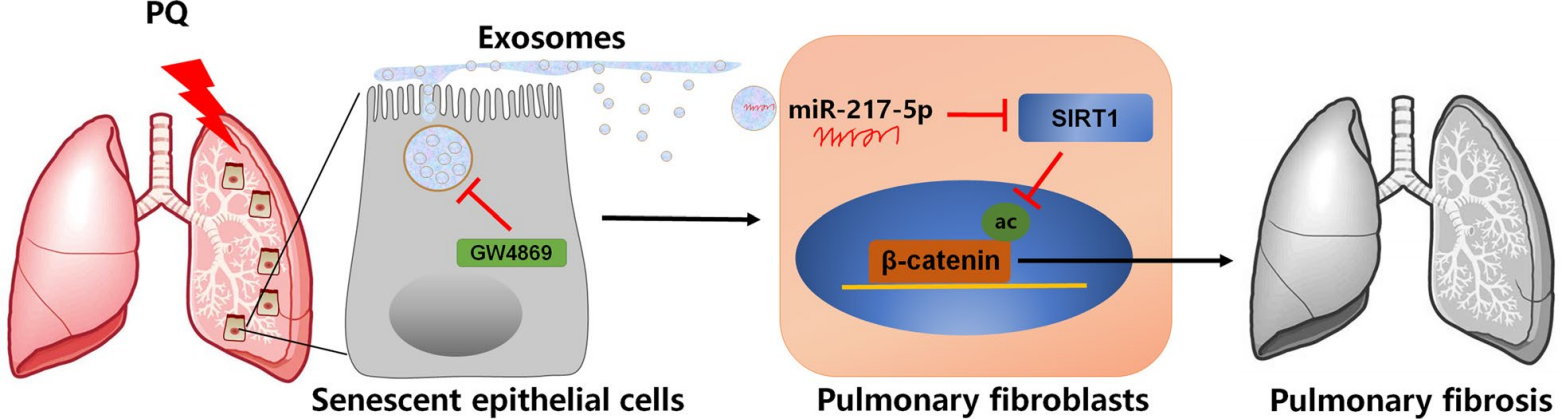
The schematic cartoon of the mechanism of PQ-mediated cellular senescence to regulate pulmonary fibroblasts activation in pulmonary fibrogenesis

To confirm whether miR-217-5p ablation could serve as a potential strategy to suppress PQ-induced pulmonary fibrogenesis in vivo, miR-217-5p antagomir was intratracheally injected into mice after PQ treatment. As expected, miR-217-5p antagomir dramatically inhibited the expression of miR-217-5p in the lung tissues of PQ-treated mice (Fig. 7A). We demonstrated that antagomir-217-5p could effectively elevate SIRT1 expression and suppress the acetylation of β-catenin in the lung tissue of PQ-poisoned mice (Fig. 7B, C). Then, the evaluation of pulmonary fibrosis was conducted using H&E and Masson's trichrome staining techniques (Fig. 7D). Meanwhile, the expression of fibrotic markers in lung tissues were profoundly enhanced after PQ treatment and obviously reversed by subsequent antagomiR-217-5p treatment (Fig. 7E, F). These findings suggest that the inhibition of miR-217-5p may confer protection against PQ-induced pulmonary fibrosis in mice Fig. 8.

## Discussion

Paraquat poisoning is a significant public health issue, commonly associated with its use as an herbicide and the potential for accidental or intentional ingestion by individuals [1]. The elevated fatality rate linked to PQ poisoning can be attributed to the progression of pulmonary fibrosis, leading to the deterioration of lung tissue and subsequent respiratory failure [29]. Previously, our study provided evidence that epithelial cells in the lung tissue of PQ-poisoned mice were predominantly in a senescent state. Furthermore, we observed that the secretion of PQ-treated epithelial cells had pro-fibrotic effects by stimulating the activation of pulmonary fibroblasts [11]. Cellular senescence is now recognized as a fundamental mechanism of aging that contributes to many diseases [30]. In vitro, senescent cells exhibit modifications in apoptotic signaling, which have a significant impact on the pathogenesis of numerous diseases by influencing the extracellular milieu and facilitating intercellular communication with neighboring cells [31, 32]. In present study, we identified the critical role of exosomes in senescent epithelial cells-mediated pulmonary fibrogenesis, which promoted the activation of pulmonary fibroblasts via transporting miR-217-5p to fibroblasts.

Exosomes, which are double-layered membrane structures secreted from cells through exocytosis, have garnered significant interest in the realm of biomedical research [33]. Indeed, exosomes play an essential role in intercellular communication by transmitting a diverse array of molecules to recipient cells. The transfer of molecules takes place through various mechanisms such as receptor-ligand interactions, direct membrane fusion, or endocytosis. This process plays a crucial role in modulating the behavior of recipient cells [34, 35]. By facilitating intercellular communication, exosomes are involved in a wide range of physiological and pathological processes, and their study would promote the development of novel therapeutic approaches in various fields of medicine. In our study, to provide additional clarification on the pro-fibrotic function of exosomes derived from PQ-treated epithelial cells, we employed the exosome secretion inhibitor GW4869 to expand upon our discoveries. These findings emphasize the crucial involvement of exosome-mediated communication between epithelial and fibroblast cells in the development of PQ-induced pulmonary fibrosis. Our findings indicate that inhibiting the release of exosomes could serve as a promising therapeutic approach for mitigating PQ poisoning.

Exosomes consistently exhibit elevated levels of miRNAs and facilitate their transfer to recipient cells. These miRNAs function as suppressors of target genes, resulting in the inhibition of translation and degradation of mRNAs [36]. Here, we found miR-21-5p, miR-192, miR-215 and miR-217-5p were significantly upregulated in the exosomes derived from PQ-treated MLE-12 cells. It was reported that exosomal miR-21 could contribute renal fibrosis via targeting PTEN [37]. miR-215 could aggravate PQ-induced pulmonary fibrosis via repressing BMPR2 [38]. In our work, we demonstrated that the presence of elevated levels of exosomal miR-217-5p plays a significant role in the activation of fibroblasts during PQ-induced pulmonary fibrogenesis. It has been reported that miR-217-5p has the ability to enhance fibrosis by regulating the expression of SIRT1 [39]. In addition, miR-217-5p has been found to be linked to senescence and angiogenesis in endothelial and smooth muscle cells by modulating the expression of SIRT1 [40]. Sirt1, a member of the silent information regulator 2 family, is widely recognized as an anti-aging molecule [41]. It has been demonstrated that SIRT1 could protect against TGF-β-induced pulmonary fibroblast activation and display anti-fibrotic effects in pulmonary fibrogenesis [42]. However, the molecular mechanism by which SIRT1 is involved in miR-217-5p-meidated fibroblasts activation is currently unknown.

It has been reported that miR-217-5p has been predicted to play a role in the regulation of Wnt signaling [43]. The activation of Wnt signaling exhibited a strong correlation with fibroblast activation and the development of pulmonary fibrosis induced by PQ [44]. β-catenin, as the key factor of Wnt signaling, was critical in modulating cell differentiation and proliferation [45]. Studies have provided evidence indicating that the upregulation and subsequent translocation of β-catenin to the nucleus can initiate the activation of cellular survival pathways [46]. The process of acetylation of β-catenin has the ability to impede the phosphorylation and ubiquitination of β-catenin, thereby facilitating its translocation into the nucleus and subsequently leading to the activation of Wnt signaling [47]. SIRT1, a NAD^+^-dependent deacetylase, is known for its high conservation across species [48], which could regulate the differentiation of mesenchymal stem cells via modulating the acetylation of d β-catenin [49]. In our work, we demonstrated that inhibition of SIRT1 can lead to an accumulation of acetylated β-catenin and an upregulation of fibrotic gene expression in pulmonary fibroblasts. Furthermore, the overexpression of miR-217-5p has been shown to increase the acetylation of β-catenin, leading to its enhanced nuclear translocation. This effect can be effectively attenuated by restoring the expression of SIRT1.

## Conclusion

Our study showed that exosomes from senescent epithelial cells promote fibrogenesis in a miR-217-5p-dependent manner, leading to the activation of fibroblasts through modulation of the SIRT1/β-catenin axis (Fig. 8). These findings suggest a novel approach for regulating exosome secretion and miR-217-5p expression, which could be used in the development of therapies for PQ poisoning.

### Supplementary Information

**Additional file1: Figure S1.** SA-β-Gal staining for MLE-12 cells treated with or without PQ. **Figure S2.** Representative images and quantification of immunofluorescent detection for CD63 in the lung tissues from mice treated with or without PQ. **Figure S3.** Representative images and quantification of immunofluorescent detection for CD63 in MLE-12 cells treated with or without PQ. **Figure S4.** The overall size distribution of exosomes isolated from the supernatant of MLE-12 cells. **Figure S5.** The representative H&E staining (up panel) and Masson’s trichrome staining (down panel) images of the lung tissues from PQ-poisoned mice injected with or without GW4869. **Figure S6.** The GFP detection (Green) in pulmonary fibroblasts co-cultured with MLE-12 cells transfected with GFP-CD63. **Figure S7.** (A) qPCR analysis of miR-215 expression in pulmonary fibroblasts co-cultured with MLE-12 cells or PQ-treated MLE-12 cells. (B) qPCR analysis of miR-215 expression in the lung tissues from mice treated with or without PQ. **Figure S8.** The position of the miR-217-5p target site in the 3′UTR region of SIRT1 was predicted via using TargetScan database. **Table S1.** Primers used for qPCR.

## Data Availability

The datasets used and/or analyzed during the present study are available from the corresponding author on reasonable request.
