# Supplementary material for: Exosomes from senescent epithelial cells activate pulmonary fibroblasts via the miR-217-5p/Sirt1 axis in paraquat-induced pulmonary fibrosis
Source: J Transl Med. 2024 Mar 26;22:310. doi: 10.1186/s12967-024-05094-x (PMC10964553; doi:10.1186/s12967-024-05094-x)
Supplement: Supplementary file 1 — Additional file1: Figure S1. SA-β-Gal staining for MLE-12 cells treated with or without PQ. Figure S2. Representative images and quantification of immunofluorescent detection for CD63 in the lung tissues from mice treated with or without PQ. Figure S3. Representative images and quantification of immunofluorescent detection for CD63 in MLE-12 cells treated with or without PQ. Figure S4. The overall size distribution of exosomes isolated from the supernatant of MLE-12 cells. Figure S5. The representative H&E staining (up panel) and Masson’s trichrome staining (down panel) images of the lung tissues from PQ-poisoned mice injected with or without GW4869. Figure S6. The GFP detection (Green) in pulmonary fibroblasts co-cultured with MLE-12 cells transfected with GFP-CD63. Figure S7. (A) qPCR analysis of miR-215 expression in pulmonary fibroblasts co-cultured with MLE-12 cells or PQ-treated MLE-12 cells. (B) qPCR analysis of miR-215 expression in the lung tissues from mice treated with or without PQ. Figure S8. The position of the miR-217-5p target site in the 3′UTR region of SIRT1 was predicted via using TargetScan database. Table S1. Primers used for qPCR. [file 12967_2024_5094_MOESM1_ESM.docx]

**Additional materials**


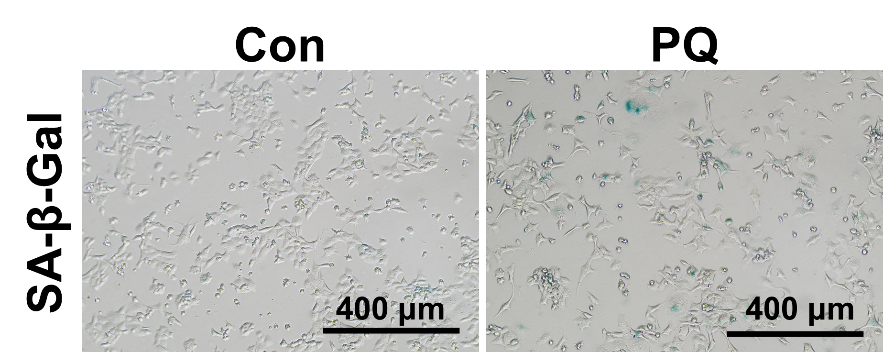


Figure S1. SA-β-Gal staining for MLE-12 cells treated with or without PQ.


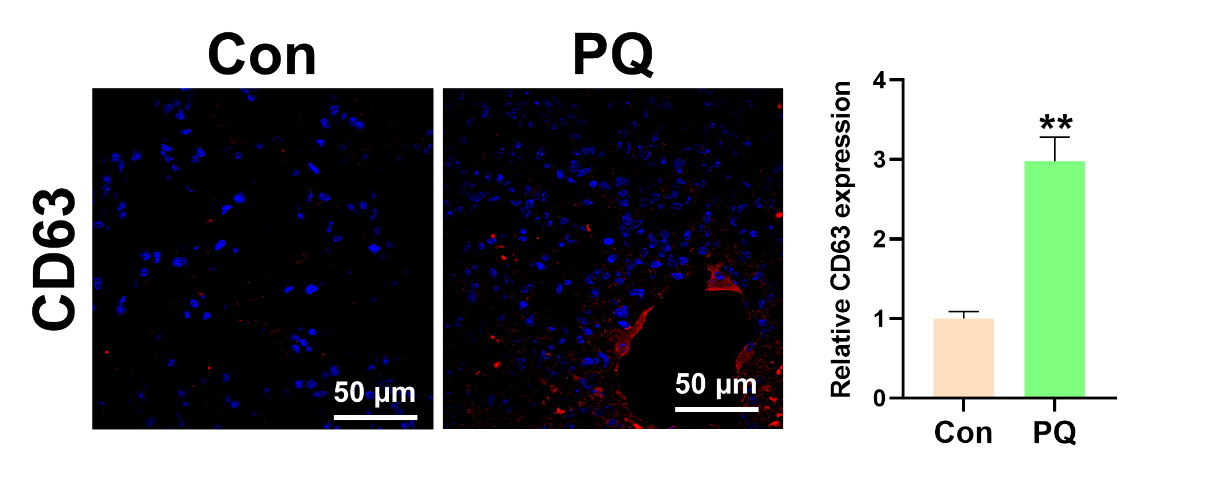


Figure S2. Representative images and quantification of immunofluorescent detection for CD63 in the lung tissues from mice treated with or without PQ.


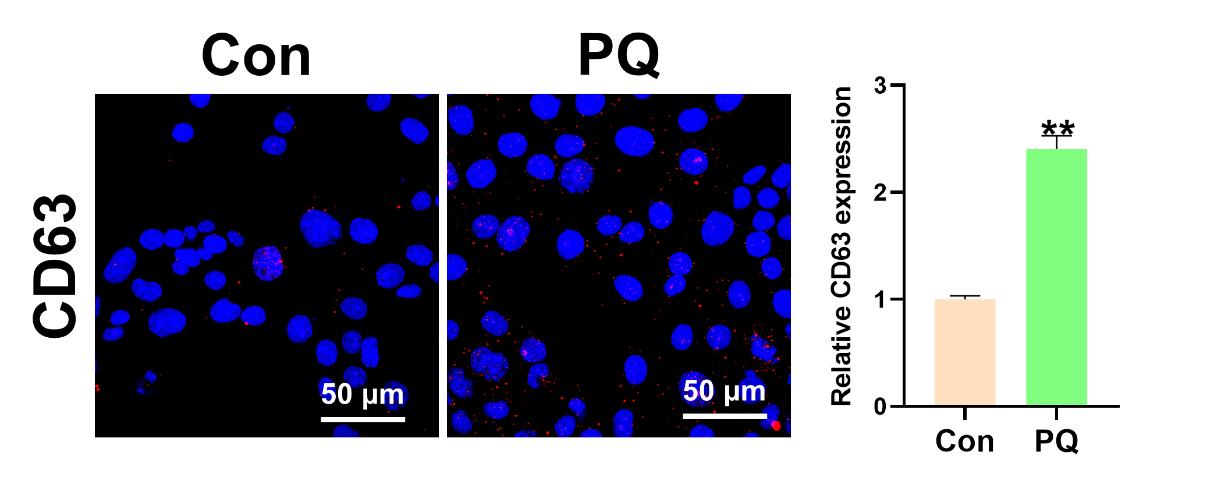


Figure S3. Representative images and quantification of immunofluorescent detection for CD63 in MLE-12 cells treated with or without PQ.

Figure S4. The overall size distribution of exosomes isolated from the supernatant of MLE-12 cells.


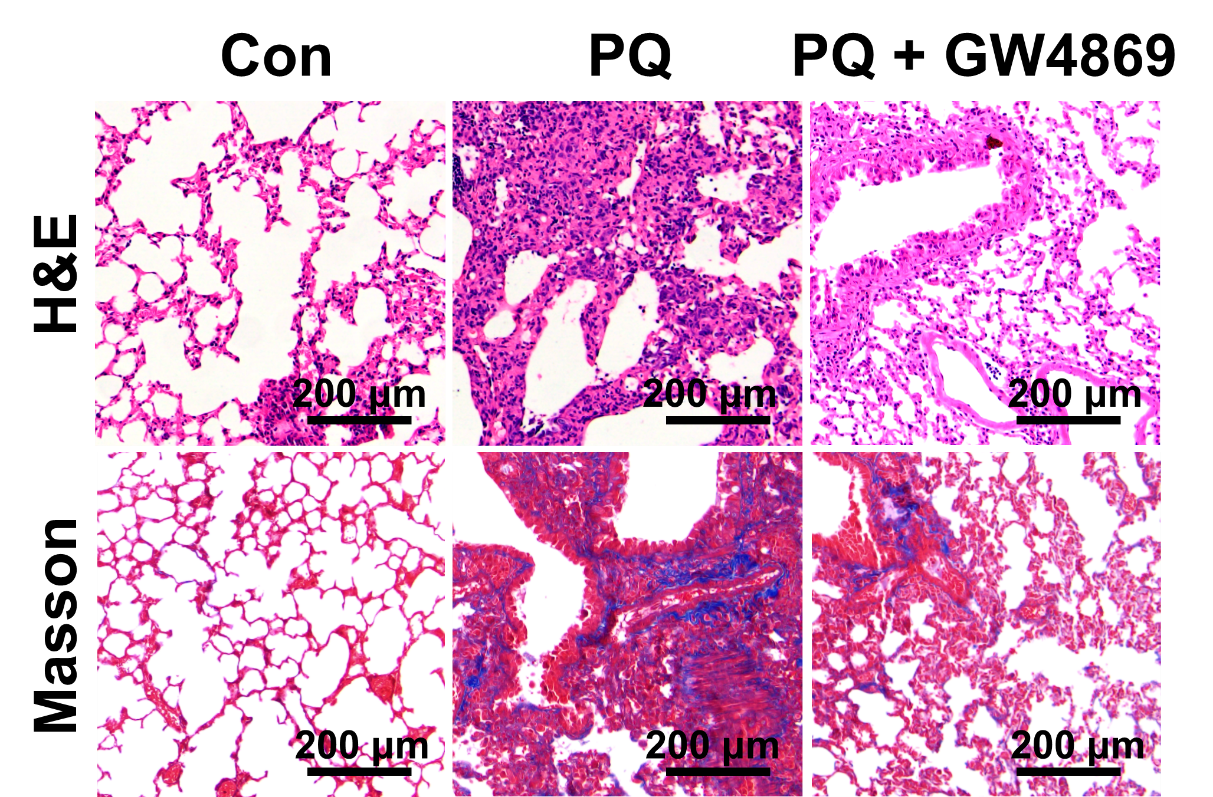


Figure S5. The representative H&E staining (up panel) and Masson’s trichrome staining (down panel) images of the lung tissues from PQ-poisoned mice injected with or without GW4869.


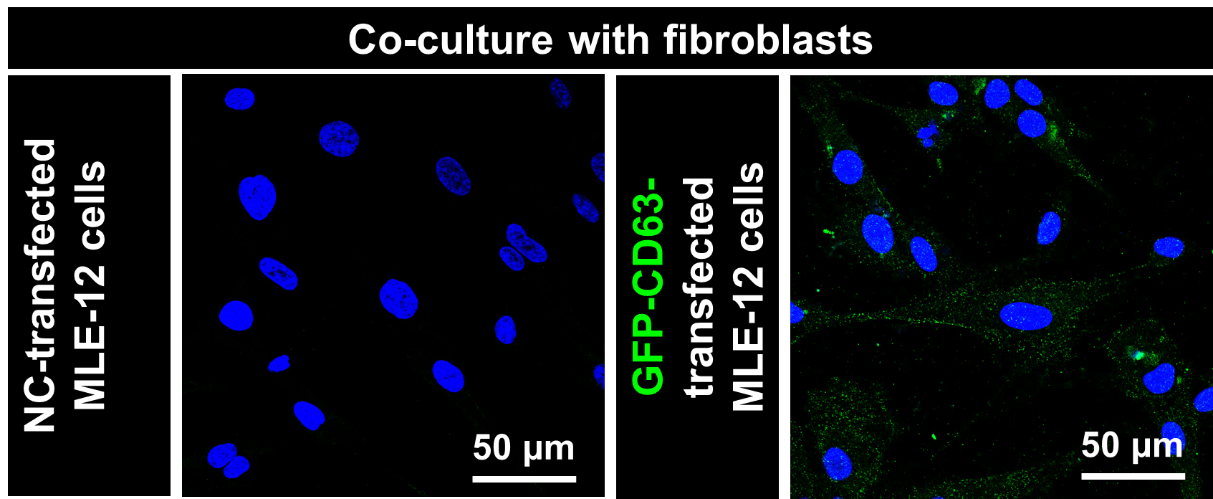


Figure S6. The GFP detection (Green) in pulmonary fibroblasts co-cultured with MLE-12 cells transfected with GFP-CD63.


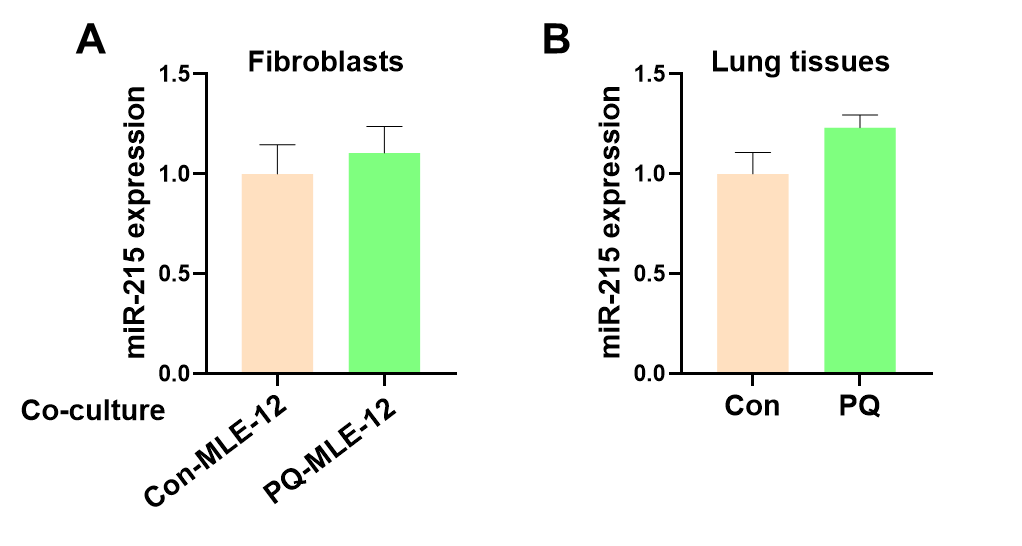


Figure S7. (A) qPCR analysis of miR-215 expression in pulmonary fibroblasts co-cultured with MLE-12 cells or PQ-treated MLE-12 cells. (B) qPCR analysis of miR-215 expression in the lung tissues from mice treated with or without PQ.


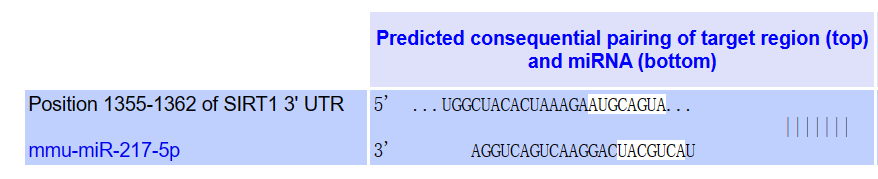


Figure S8. The position of the miR-217-5p target site in the 3′-UTR region of SIRT1 was predicted via using TargetScan database.


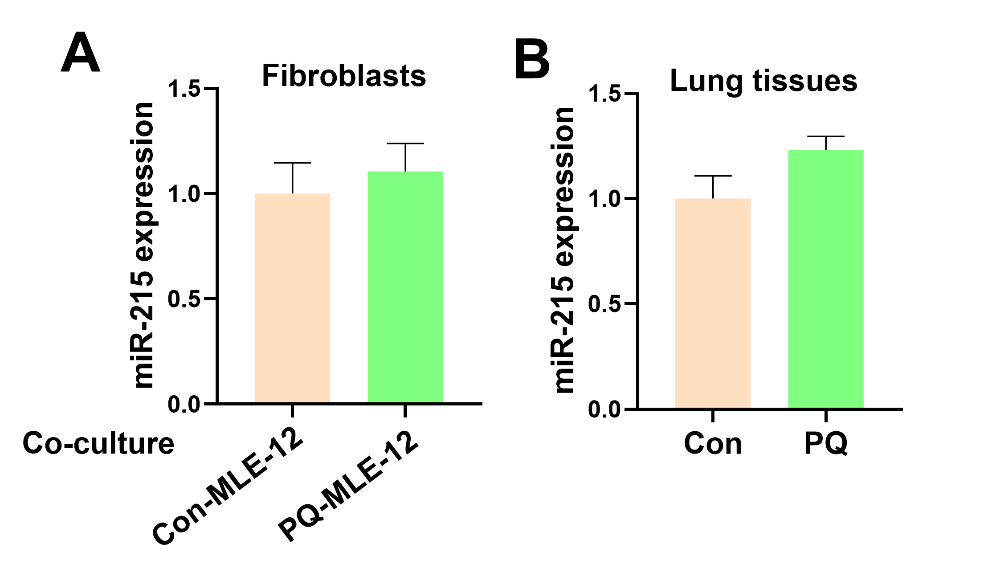


**Table S1.** Primers used for qPCR.

|  | Primer sequence (5’-3’) | |
| --- | --- | --- |
|  |  | |
| Target Gene | Forward | Reverse |
| *Acta2* | GTCCCAGACATCAGGGAGTAA | TCGGATACTTCAGCGTCAGGA |
| *Col1a1* | GCTCCTCTTAGGGGCCACT | CCACGTCTCACCATTGGGG |
| *Sirt1* | GCTGACGACTTCGACGACG | TCGGTCAACAGGAGGTTGTCT |
| *Gapdh* | AGGTCGGTGTGAACGGATTTG | TGTAGACCATGTAGTGAGGTCA |
